# The role of Ca^2+^/NFAT in Dysfunction and Inflammation of Human Coronary Endothelial Cells induced by Sera from patients with Kawasaki disease

**DOI:** 10.1038/s41598-020-61667-y

**Published:** 2020-03-13

**Authors:** Ying Wang, Jian Hu, Jingjing Liu, Zhimin Geng, Yijing Tao, Fenglei Zheng, Yujia Wang, Songling Fu, Wei Wang, Chunhong Xie, Yiying Zhang, Fangqi Gong

**Affiliations:** Department of Cardiology, Children’s Hospital, Zhejiang University School of Medicine, National Clinical Research Center for Child Health, No. 3333 Binsheng Road, Hangzhou, 310052 PR China

**Keywords:** Paediatric research, Cardiovascular diseases

## Abstract

Ca^2+^/nuclear factor of activated T-cells (Ca^2+^/NFAT) signaling pathway may play a crucial role in the pathogenesis of Kawasaki disease (KD). We investigated the poorly understood Ca^2+^/NFAT regulation of coronary artery endothelial cells and consequent dysfunction in KD pathogenesis. Human coronary artery endothelial cells (HCAECs) stimulated with sera from patients with KD, compared with sera from healthy children, exhibited significant increases in proliferation and angiogenesis, higher levels of NFATc1 and NFATc3 and some inflammatory molecules, and increased nuclear translocation of NFATc1 and NFATc3. HCAECs stimulated with sera from patients with KD treated with cyclosporine A (CsA) showed decreased proliferation, angiogenesis, NFATc1 and inflammatory molecules levels as compared with results for untreated HCAECs. In conclusion, our data reveal that KD sera activate the Ca^2+^/NFAT in HCAECs, leading to dysfunction and inflammation of endothelial cells. CsA has cytoprotective effects by ameliorating endothelial cell homeostasis via Ca^2+^/NFAT.

## Introduction

Kawasaki Disease (KD), also known as mucocutaneous lymph node syndrome (MCLS), is an acute febrile rash that occurs mainly in children under 5 years which is the most common systemic vasculitis syndrome in children^1^. The disease mainly affects small and medium arteries, especially coronary arteries. Coronary artery lesions (CAL) can occur in 25% of patients^2^. Coronary artery injury and coronary aneurysm formation are the most important acute and long-term sequela of KD^3,4^.

The pathogenesis of KD remains unclear. Recent studies have confirmed that vascular endothelial cells (VECs) play a very important role in the coronary artery injury of KD^5^. Children with KD have abnormal activation of the immune system; VECs are stimulated to express and release a variety of adhesion factors, which in turn cause inflammatory cells to adhere to the surface of endothelial cells, which resulting in inflammation to damage of VECs^6^. The dysfunction of VECs plays a vital role in vascular tension, lipid metabolism and coagulation mechanism, which in turn activate inflammatory cells, causing inflammation extending deep into the endothelium, leading to destruction of the vascular elastic layer, formation of coronary aneurysm and vascular remodeling^7^.

The nucleus factors of activated T cells (NFAT), initially found in activated T cells, are widely expressed in mammalian cells^8^. The role of NFAT signaling is not limited to the immune system: NFAT protein is involved in various genes regulation, which regulates the development and differentiation of mammalian cells and tissues^9^. Activation of the Ca^2+^/NFAT signaling pathway is associated with angiogenesis and inflammation in endothelial cells^10,11^. Studies have shown that the Ca^2+^/NFAT pathway is the main signaling pathway of vascular endothelial growth factor (VEGF) stimulating endothelial cells^12^. In VECs, VEGF-induced Down Syndrome Critical Region (DSCR)-1 acts as a negative feedback loop to inhibit NFAT-mediated proliferation and activation of ECs^13,14^. Consequently, the Ca^2+^/NFAT signaling pathway plays an important role in maintaining normal structure and function of VECs.

In addition, genetic analysis revealed mutations in 16 sites of key genes of Ca^2+^/NFAT signaling pathway in children with KD; and the mutant rs1561876AA is strongly correlated with vasculitis in KD^15,16^. These findings further suggest that the Ca^2+^/NFAT signaling pathway plays an important role in KD coronary artery injury. At present, various KD drugs targeting the Ca^2+^/NFAT signaling pathway have been reported, for example, cyclosporine A (CsA) and FK506 are NFAT-targeted drugs^17^. However, the molecular mechanism of Ca^2+^/NFAT signaling pathway in KD coronary artery injury remain unclear.

Here, we developed an *in vitro* method to characterize the function of Ca^2+^/NFAT in human coronary artery endothelial cells during acute KD. Referring to the previously reported method, Ueno *et al*. and He *et al*. used sera from patients with KD to stimulate human umbilical vein endothelial cell line, inducing cellular immune damage, to achieve KD vasculitis^18,19^. However, there have been no reports of sera from patients with KD stimulating human coronary endothelial cells.

Thus, we hypothesized that Ca^2+^/NFAT may plays a key role in the pathogenesis of KD vasculitis. Herein, we focused on the pathogenesis of early KD vasculitis by examining the dysfunction and inflammation of endothelial cells. For characterizing the function of Ca^2+^/NFAT in human coronary artery endothelial cells (HCAECs), we firstly used sera from patients with KD to stimulate *in vitro* HCAECs, and analyzed whether Ca^2+^/NFAT are activated in the development of KD. Furthermore, we investigated the therapeutic effects of CsA on HCAECs stimulated with sera from patients with KD.

## Results

### Levels of NFAT mRNA and protein were higher in the KD group

**Figure 1:**
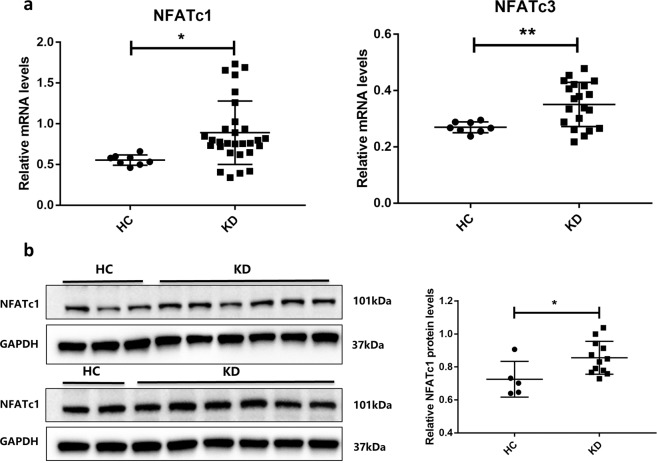
Levels of NFAT mRNA and protein were higher in the KD group. (**a**,**b**) HCAECs were treated with medium containing 15% sera from HC (n = 8) and KD (n = 29) subjects. After 6 hours, NFATc1 and NFATc3 mRNA levels were measured by qPCR (**a**: all samples were normalized to HCAECs with 15% FBS treatment), and protein levels in HCAECs were measured by Western blot (**b**; HC, n = 5 and KD, n = 12). The NFATc1 and GAPDH blots in one group were cropped from different parts of the same gel. The upper group and lower group of blots were cropped from different gels. The right panel of (**b**) is the statistical analysis of immunoblots. NFATc1 protein levels were measured by Western blotting. Full-length blots are presented in Supplementary Fig. S1. The bar graphs are mean ± SEM. *P < 0.05 and **P < 0.01 compared with respective control group.

We first compared the level of NFAT family mRNA and proteins in coronary artery endothelial cells when treated with KD sera. NFATc1 mRNA levels in HCAECs treated with KD sera were higher than those treated with healthy control (HC) sera (n = 29, median 0.89 vs. n = 8, median 0.55, *P* = 0.0212) (Fig. 1a). Meanwhile, NFATc3 mRNA levels were also higher in HCAECs treated with KD sera when compared with HC (n = 29, median 0.35 vs. n = 8, median 0.27, *P* = 0.0084) (Fig. 1a). The expression level of NFATc1 protein was higher in KD than in HC (n = 12, median 0.86 vs. n = 5, median 0.73, *P* = 0.0296) (Fig. 1b).

### Ca^2+^/NFAT was activated in coronary artery endothelial cells induced by KD sera

**Figure 2:**
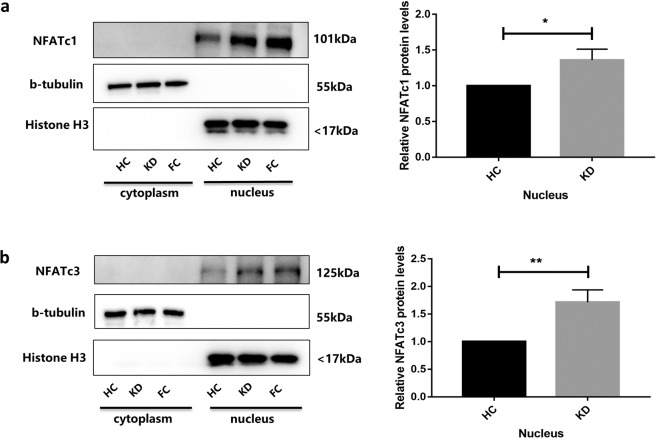
Ca ^2+^/NFAT were activated in coronary artery endothelial cells induced by KD sera. (**a**,**b**) HCAECs were incubated with HC sera (pooled from 8 individuals), KD sera (pooled from 12 individuals) and febrile control (FC) sera (pooled from 10 individuals) for 6 hours. The cytoplasm proteins and nucleus proteins were extracted by Nuclear and Cytoplasmic Protein Extraction Kit. Levels of NFATc1 and NFATc3 proteins were measured using Western blotting (in **a** and **b**). The NFATc1, b-tubulin and Histone H3 blots in one group were cropped from different parts of the same gel. Full-length blots are presented in Supplementary Fig. S1. Data in (**a**,**b**) are mean ± SEM from 3 independent experiments involving different batches of cells but the s ame pooled HC or KD sera. *P < 0.05 and **P < 0.01 when compared with respective controls or between indicated groups.

HCAECs were incubated with HC sera (pooled from 8 individuals) or KD sera (pooled from 12 individuals) for 6 hours. The nucleus protein levels of NFATc1 were higher in KD than in HC (n = 3, median 1.36 vs. n = 3, median 1, *P* = 0.0143), and nucleus protein levels of NFATc3 were also higher in KD than in HC (n = 3, median 1.71 vs. n = 3, median 1, *P* = 0.0051) (Fig. 2a,b).

### Disruption of endothelial cell homeostasis in the KD group

**Figure 3:**
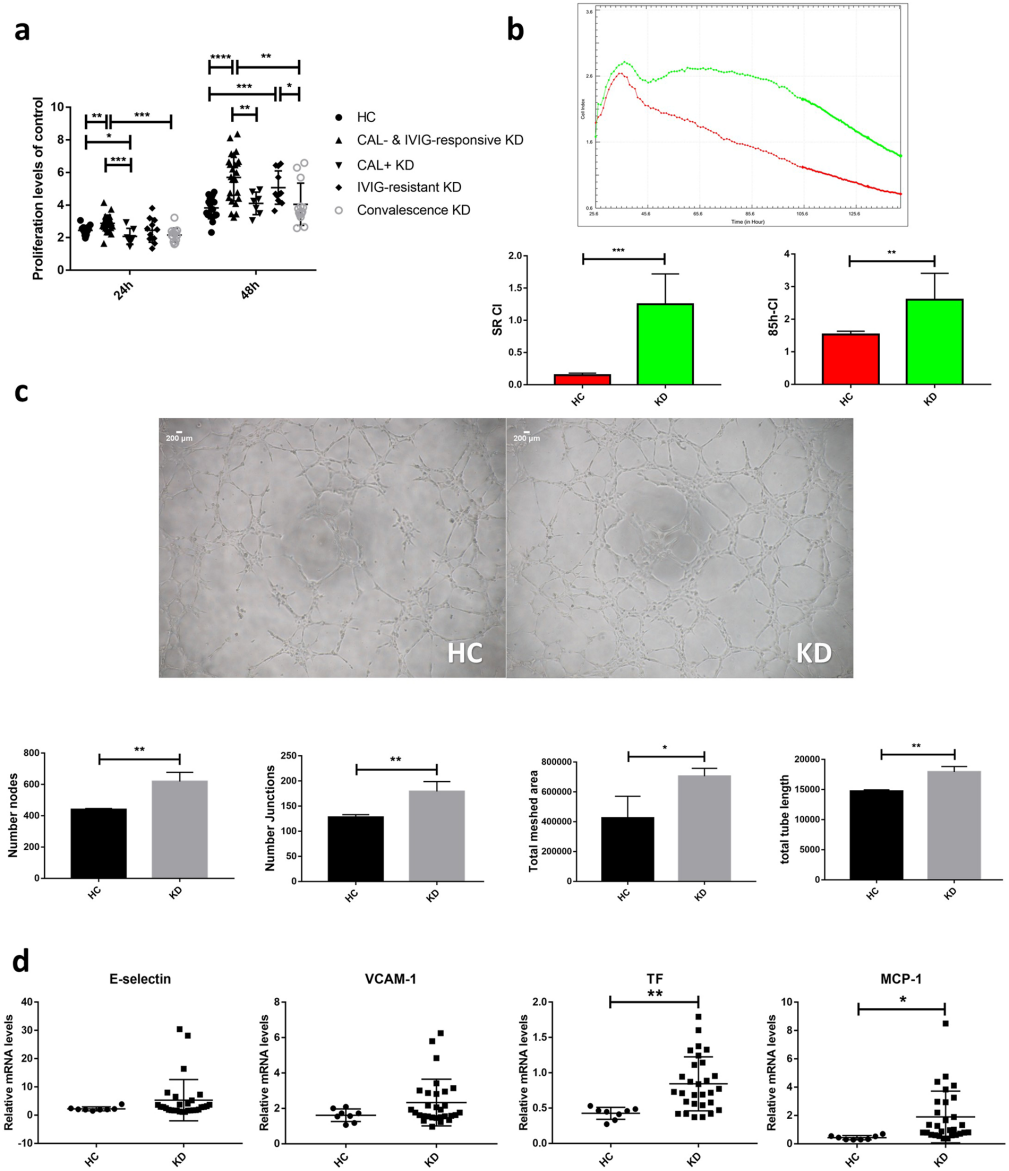
Disruption of endothelial cell homeostasis in the KD group. (**a**) HCAECs were treated with medium containing 15% sera from HC (n = 17), CAL- and IVIG-responsive KD (n = 25), CAL+ KD (n = 7), IVIG-resistant KD (n = 11) and convalescence KD (n = 13) subjects. After 24 hours and 48 hours, the proliferation of HCAECs were detected by CCK8 (all samples were normalized to HCAECs with 15% FBS treatment and each sample was examined in triplicate). (**b**) HCAECs were treated with medium containing 15% sera from HC (n = 3) and KD (n = 8) subjects. Cells proliferation was monitored for 140 h by RT-CES system. Green curve and red curve represent mean cell numbers of KD group and HC group respectively. SR CI indicates slope rate cell index; 85h-CI indicates cell index at 85 h. (**c**) *In vitro* angiogenesis. HCAECs were plated on growth factor-reduced Matrigel to migrate or join together in the presence of 15% sera from HC (pooled from 8 individuals) and KD (pooled from 12 individuals) subjects. After 4 hours, cells were photographed at a magnification of 40×. The lower panel of (**c**) is the statistical analysis of angiogenesis. (**d**) HCAECs were treated with medium containing 15% sera from HC (n = 8) and KD (n = 29) subjects. After 6 hours, E-selectin, VCAM-1, TF and MCP-1 mRNA levels were measured by qPCR (all samples were normalized to HCAECs with 15% FBS treatment). The bar graphs are mean ± SEM. *P < 0.05, **P < 0.01, ***P < 0.001 and ****P < 0.0001 compared with respective control group.

Sera from individuals to stimulate HCAECs and detected proliferation after 24 h and 48 h incubation. As shown in the Fig. 3a, the proliferation of HCAECs in the CAL- & IVIG-responsive KD group was higher than the HC and convalescence KD group at 24 h (n = 25, median 2.88 vs. n = 17, median 2.45, *P* = 0.0027; n = 25, median 2.88 vs. n = 3, median 2.16, *P* = 0.0001, respectively) and at 48 h (n = 25, median 5.69 vs. n = 17, median 3.83, *P* < 0.0001 and n = 25, median 5.69 vs. n = 13, median 4.05, *P* = 0.0014, respectively). The proliferation of HCAECs in the CAL+KD group was lower than the CAL- & I VIG-responsive KD group at 24 h (n = 7, median 2.08 vs. n = 25, median 2.88, *P* = 0.0009) and at 48 h (n = 7, median 4.10 vs. n = 25, median 5.69, *P* = 0.0086). There is no significant difference between the CAL- & IVIG-responsive KD group and IVIG-resistant KD group (*P* > 0.05) (Fig. 3a). HCAECs were detected by real-time cell electronic sensing assay (RTCA) when they were cultured with sera from patients with HC and KD. RTCA results showed that multiplication rate of KD group was higher than HC group at early stage (n = 8, median 1.25 vs. n = 4, median 0.15, *P* = 0.0003). At 85 h, the number of HCAECs in the KD group was higher than that in the HC group (n = 8, median 2.6 vs. n = 4, median 1.53, *P* = 0.0071) (Fig. 3b).

The angiogenesis ability of HCAECs in the KD group was enhanced when compared with the HC group (Number nodes, median 619 vs. 440, *P* = 0.0036; number junctions, median 179.25 vs. 128, *P* = 0.0074; total meshes area, median 705486 vs. 425797, *P* = 0.0150; total tube length, median 17923.8 vs. 14746, *P* = 0.0020) (Fig. 3c). The mRNA levels of tissue factor (TF) (median 0.84 vs. 0.43, *P* = 0.0044) and C-C motif chemokine 2 (MCP-1) (median 1.89 vs. 0.43, *P* = 0.0317) in HCAECs were higher after KD sera stimulus than the HC group. E-selectin and vascular cell adhesion protein 1 (VCAM-1) mRNA levels were higher in the KD group than the HC group, but without significant difference (E-selectin, median 5.30 vs. 2.24, *P* = 0.2493; VCAM-1, median 2.33 vs. 1.61, *P* = 0.1394) (Fig. 3d).

### CsA inhibited inflammation and attenuated dysfunction via Ca^2+^/NFAT

**Figure 4:**
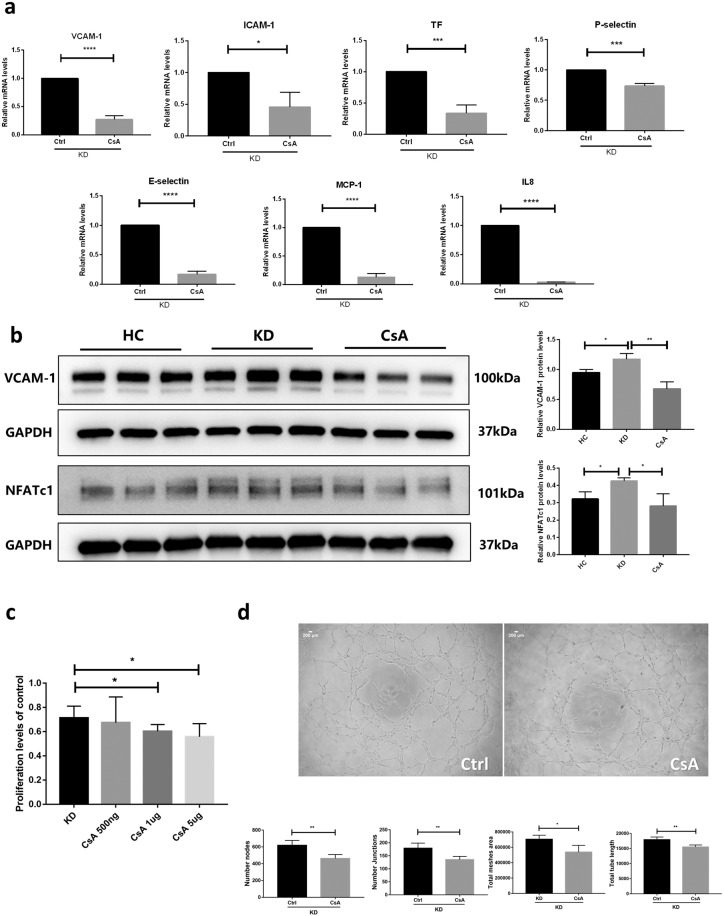
CsA inhibited inflammation and attenuated dysfunction via Ca^2+^/NFAT. (**a**,**b**) HCAECs were pretreated with KD sera (pooled from 12 individuals) or HC sera (pooled from 8 individuals) for 6 hours and treated with or without CsA (500 ng/ml) for 2 h. VCAM-1, ICAM-1, TF, P-selectin, E-selectin, MCP-1 and IL8 mRNA and protein were detected and quantified. (**b**) The VCAM-1 and GAPDH blot in one group were cropped from different parts of the same gel. The NFATc1 and GAPDH blot in one group were cropped from different parts of the same gel. Full-length blots are presented in Supplementary Fig. S1. (**c**) HCAECs were pretreated with KD sera (pooled from 12 individuals) for 24 hours and treated with different concentration of CsA for 24 h. Cells proliferation were detected and quantified. (**d**) HCAECs were plated on Matrigel in the presence KD sera (pooled from 12 individuals) or co-cultured with KD sera and CsA. After 4 hours, cells were photographed and quantified. Data are mean ± SEM from 3 independent experiments. *P < 0.05, **P < 0.01, ***P < 0.001 and ****P < 0.0001 compared with the control or indicated group.

Incubating HCAECs with KD sera increased the expression of inflammation markers, including E-selectin, VCAM-1, TF and MCP-1 (Fig. 3d). In contrast, after incubation of CsA, expression levels of VCAM-1, intercellular adhesion molecule 1 (ICAM-1), TF, P-selectin, E-selectin, MCP-1 and interleukin-8 (IL-8) were remarkably decreased (VCAM-1, median 0.27 vs. 1, *P* < 0.0001; ICAM-1, median 0.46 vs. 1, P = 0.0157; TF, median 0.34 vs. 1, *P* = 0.0009; P-selectin, median 0.73 vs. 1, *P* = 0.0004; E-selectin, median 0.17 vs. 1, *P* < 0.0001; MCP-1, median 0.13 vs. 1, *P* < 0.0001 and IL-8, median 0.03 vs. 1, *P* < 0.0001) (Fig. 4a). The protein levels of VCAM-1 and NFATc1 were higher in the KD group than those in the HC group (VCAM-1, median 1.17 vs. median0.95, *P* = 0.0205; NFATc1, median 0.43 vs. median 0.32, *P* = 0.0154, respectively), which can be inhibited by CsA (VCAM-1, median 0.68 vs. median 1.17, *P* = 0.0043; NFATc1, median 0.28 vs. median 0.43, *P* = 0.0253, respectively) (Fig. 4b). Co-incubation of KD sera and CsA induced the decrease of endothelial cells proliferation. With the CsA concentration increasing, the inhibition ability was more obvious (CsA 1 ug/ml, median 0.60 vs. 0.71, *P* = 0.0356; CsA 5 ug/ml, median 0.56 vs. 0.71, *P* = 0.0256) (Fig. 4c). Furthermore, CsA co-incubation with KD sera reduced the angiogenesis ability (Number nodes, median 462.75 vs. 619, *P* = 0.0059; number junctions, median 135.25 vs. 179.25, *P* = 0.0091; total meshes area, median 538233 vs. 705486, *P* = 0.0168; total tube length, median 1 5538.3 vs. 17923.8, *P* = 0.0052) (Fig. 4d).

## Discussion

The main finding of this study is the activation of Ca^2+^/NFAT in human coronary endothelial cells after incubated with sera from patients with KD. Compared with the controls, endothelial cells treated with KD sera had increased expression levels of NFATc1, NFATc3 and inflammatory molecules (like E-selectin, VCAM-1, TF and MCP-1). Meanwhile, the function of endothelial cells was disrupted in treated groups, which had increased proliferation and enhanced angiogenesis. This disruption of endothelial cell homeostasis likely affected vascular wall injury and aneurysm formation in acute KD. Furthermore, our results are based on the fact that cyclosporine A can specifically inhibit Ca^2+^/NFAT. Thus, CsA suppressed the inflammation and attenuated dysfunction in coronary endothelial cells via Ca^2+^/NFAT.

At present, research hotspots on the pathogenesis of KD are mainly divided into two aspects including inflammatory immune mechanism and coronary vascular endothelial cell injury. Due to the obstacle in the isolation of primary coronary endothelial cells in mice, most studies on coronary artery injury have been carried out in umbilical vein endothelial cells. However, venous endothelial cells are different from arterial endothelial cells. For instance, Li *et al*. found Aryl hydrocarbon receptor (AhR), a ligand-activated transcription factor, involved in regulation of vascular development and angiogenesis, which plays differential roles in human artery and vein endothelial cells^20^. In addition, endothelial cells from umbilical arteries (HUAEC) and vein (HUVEC) have differential modulation of biologically active sex steroid levels, with apparent higher inactivation in the arterial system^21^.

KD is a systemic vasculitis characterized by activation of arterial endothelial cells. The expression of adhesion molecules such as VCAM-1, is augmented by cytokines. These adhesion molecules participate in the migration of leukocytes to sites of inflammation, enabling firm adhesion and diapedesis of leukocytes^22^. Studies have reported that adhesion molecules are involved in the pathogenesis of KD^23–25^. Levels of soluble E-selectin are elevated in sera during acute KD^26^. In our study, we found increased VCAM-1 of HCAECs in the KD group, similar to the previous report^27^. In addition, MCP-1 is a potent monocyte chemoattractant protein which is responsible for the recruitment of mononuclear cells at the site of KD lesions^28^. We also demonstrated that MCP-1 and other inflammatory molecules, like TF, were all elevated in HCAECs after stimulated with KD sera. Thus, HCAECs are activated by sera components from KD patients and manufacture a series of inflammatory mediators, thereby resulting in inflammatory cascade reaction that may lead to CAL in KD.

In order to study the functions of HCAECs stimulated after KD sera, we detected cell proliferation and angiogenesis. We found that the proliferation of HCAECs in the CAL- & IVIG-responsive KD group at 24 h and 48 h were significantly higher than that in the HC group by CCK-8 assay. The previously reported results are conflicting. Hashimoto *et al*. and Higashi *et al*. found that KD sera enhanced HUVECs proliferation^29,30^. Circulating immune complex in KD sera were negative, which suggesting that the enhanced endothelial cell proliferation was induced by sera components other than immune complexes^30^. On the contrary, Ueno *et al*. and Wu *et al*. demonstrated that KD sera resulted in HUVECs increased cytotoxicity and apoptotic effects^19,31^. The enhanced proliferation of HCAECs induced by KD sera in our study were due to the following reasons: (1) In the acute phase of KD, coronary endothelial cells are activated to show enhanced proliferation ability to cope with environmental changes, which is a feedback regulation to stress^32^; (2) The sera composition is more complicated. VEGF concentration was reported to be high in KD sera which promotes endothelial proliferation^33–35^; (3) The proliferation of endothelial cells is an early response to endothelial-mesenchymal changes^36^. Interestingly, we found that HCAECs incubated with sera from CAL+ KD patients did not show increased proliferation. We speculate that the CAL+ patients were delayed diagnosed when they arrived in hospital and sera collected from CAL+ patients in late illness day contained low concentration of inflammatory factors. In order to rule out the instability of CCK8 absorbance, we detect the cell proliferation ability under the same conditions by RTCA and obtained the same results. Due to the heterogeneity in sera composition in patients with KD, it requires a more detailed classification and a larger sample size of patient sera. We then examined angiogenesis changes of coronary endothelial cells in the constructed model. Higashi *et al*. found that sera from patients with KD (especially coronary aneurysms) was less active in stimulating HUVEC tube formation than sera from healthy controls or febrile controls^29^. Unlike the previous report, our data reveal the angiogenesis ability of HCAEC was enhanced. The enhanced sera angiogenesis activity was partly caused by the increase in the sera activity of stimulating HCAECs proliferation.

In our study, levels of NFATc1 and NFATc3 mRNAs and NFATc1 protein were higher in the KD group. The nucleus proteins of NFATc1 and NFATc3 were also higher in the KD group than the HC group. We demonstrated that sera from KD patients induced increased Ca^2+^/NFAT activity in activated endothelial cells, as compared with sera from healthy patients. The Ca^2+^/NFAT signaling pathway is a crucial signaling pathway for maintaining the normal physiological functions of the cardiovascular system as well as pathophysiology. Studies have reported that the Ca^2+^/NFAT signaling pathway is essential for coronary angiogenesis and venous valves development during vascular plexus formation^37–39^. Reports have shown that the NFAT pathway may be the main signaling pathway of vascular endothelial growth factor (VEGF) stimulating on endothelial cells^40^. Meanwhile, VEGF content in the KD sera is higher^33–35^. Thus, we infer that KD sera may induce elevated proliferation and enhanced angiogenesis in HCAECs via activated Ca^2+^/NFAT. Five isoforms of NFAT (NFATc1 to NFATc5) have been cloned to date, with NFAT5 lacking the corresponding sites for binding to calcineurin (CaN) and NFATc1 to NFATc4 being expressed in the cardiovascular system^41^. In the vascular system NFAT (including the isoforms c1 and c3) contributes to cell growth^42,43^, controls vascular development and angiogenesis^44,45^ and is activated in response to inflammatory processes^46^. Furthermore, the regulation of nuclear localization of NFAT is isoform-specific. Activation of NFATc1 is persistent nuclear localization, NFATc3 is only transiently imported into the nucleus, followed by rapid export back to the cytoplasm^47^. In our study, we mainly found the activation of NFATc1 and NFATc3. In previous studies, researchers found that some genes in the Ca^2+^/NFAT signaling pathway are mutated in KD patients, and the role of this pathway in vascular biology suggests that the Ca^2+^/NFAT signaling pathway may be associated with KD^15,16,48^. Kishi *et al*. found a functional SNP (itpkc_3) in the inositol 1,4,5-trisphosphate 3-kinase C (*ITPKC*) gene that is significantly associated with KD susceptibility and incidence of CAL. The C allele of itpkc_3 reduces splicing efficiency of the *ITPKC* mRNA, and ITPKC acts as a negative regulator of T-cell activation through the Ca^2+^/NFAT signaling pathway^48^. We proposed following the pathological process of KD via dysfunction and inflammation of arterial endothelial cells. Ca^2+^/NFAT is hyper-activated in the patients with KD because of the genetic susceptibility and inflammatory molecules downstream of this pathway are secreted into sera. Sera components from KD patients (may be VEGF or other inflammatory molecules) induce activation of Ca^2+^/NFAT in endothelial cells and dysfunction of endothelial cells, which may also contribute to the development of vascular complications in KD.

We observed that after Ca^2+^/NFAT pathway-specific inhibitor CsA incubation, the expression of inflammatory molecules (like E-selectin, VCAM-1, TF and MCP-1) downstream of this pathway is significantly inhibited. Meanwhile, CsA can ameliorate the dysfunction of endothelial cells induced by KD sera. At present, various KD drugs targeting Ca^2+^/NFAT signaling pathway have been reported, for example, cyclosporine A (CsA) and FK506 are NFAT-targeted drugs. CsA has been used safely in treating IVIG-resistant Japanese patients with KD^49^. Tremoulet *et al*. reported that CaN inhibitors were efficacious and well-tolerated in 10 IVIG-resistant KD patients (9 with CSA and 1 with tacrolimus)^50^. Moreover, CsA treatment can effectively reduce the persisting sera inflammatory cytokines in most of the IVIG-resistant KD patients^51^. Thus, CsA may represent a promising agent for the treatment of KD. Our results approved that dysfunction and inflammation in HCAECs induced by KD sera were mainly via Ca^2+^/NFAT signaling way. Our study also provides novel insights into the cytoprotective properties of CsA in KD vasculitis, specifically with respect to the regulation of cell dysfunction and inflammation in response to vascular homeostasis.

In conclusion, our data revealed that Ca^2+^/NFAT was responsible for dysfunction and inflammation in activated endothelial cells induced by sera from KD patients, thereby contributing to the pathogenesis of KD vasculitis. CsA treatment plays an important role in cytoprotection, which can ameliorate endothelial cell dysfunction and inflammation in vasculitis cause by KD. NFAT may provide a new marker for clinical prediction of the occurrence of KD, and inhibitors against NFAT are also expected to provide new ideas and drugs for clinical treatment of KD.

## Methods

### Human subjects

The study protocol was approved by the affiliated Children’s Hospital of Zhejiang University Ethical Committee and was performed in accordance with the Declaration of Helsinki. All KD patients and healthy children were included after obtaining written informed consent from their parents. The demographic and clinical characteristics of the subjects involved in the present study are presented in Table 1. All patients diagnosed with KD, CAL and IVIG-resistant KD met the 2017 AHA diagnostic criteria^52^. In the present study, the sera of 60 acute KD children, 17 healthy children, 14 convalescent KD children and 10 febrile children were collected from the affiliated Children’s Hospital of Zhejiang University (Hangzhou, China) between April 2018 and May 2019. Among the acute KD patients, 7 had CAL and 11 were IVIG-resistant (Table 2). All sera from acute KD patients are before IVIG treatment. All sera samples were filtered and stored at −80 °C until usage.

**Table 1:** Characteristics of the clinical index independent validation cohort in HC, acute KD and Convalescence KD.

Group	HC (n = 17)	Acute KD (n = 60)	Convalescence KD (n = 14)
Male, N	9	34	8
Age at onset (years)	4.2(2.3–5.5)	2.3(1.1–3.5)	2.3(0.8–4.0)
Body weight (kg)	16.4(13.0–21.0)	13.2(9.5–15.0)	12.2(9.5–15.6)
Illness days	/	6.3(4.0–6.0)	18.9(13.3–23.5)
WBC (*109/L)	8.0(6.8–8.6)	12.5(10.3–15.0)	10.5(6.1–13.3)
#N (*109/L)	3.1(2.3–3.4)	7.7(4.5–9.7)	7.0(3.6–6.7)
Plt (*109/L)	298.5(284.0–321.0)	364.4(273.0–416.5)	354.9(266.3–436.3)
ESR (mm/h)	/	55.4(35.0–71.0)	/
CRP (mg/dl)	<0.5	68.2(36.7–89.3)	<0.5

Data are expressed as median values and interquartile range (25th, 75th percentile), or number. KD, Kawasaki disease; HC, health control; Illness days, illness days before sample collection; WBC, white blood cell; #N, absolute value of neutrophil; Plt, platelet; ESR, erythrocyte sedimentation rate; and CRP, C-reactive protein.

**Table 2:** Characteristics of the clinical index independent validation cohort in CAL- & IVIG-responsive KD, CAL and IVIG-resistant KD.

Group	CAL- & IVIG-responsive (n = 42)	CAL+ (n = 7)	IVIG-resistant (n = 11)
Male, N	23	5	6
Age at onset (years)	2.3(0.8–3.7)	2.3(1.7–2.8)	2.3(1.3–3.3)
Body weight (kg)	13.4(9.6–14.9)	12.6(11.3–14)	12.6(9.5–15.0)
Illness days	5.5(4.0–6.0)	12.9(6.5–16.5)	5.1(4.0–6.0)
WBC (*109/L)	12.7(10.6–15.1)	11.2(8.1–12.1)	12.5(7.9–15.3)
#N (*109/L)	7.7(4.9–9.4)	5.4(3.0–6.3)	8.7(3.6–11.9)
Plt (*109/L)	335.0(268.0–379.0)	574.5(462.0–620.0)	359.3(330.5–385.5)
ESR (mm/h)	56.1(34.3–77.3)	52.3(36–62.3)	54.2(40.5–68.0)
CRP (mg/L)	69.9(35.0–89.0)	52.0(54.3–60.0)	69.3(54.8–94.6)
Z-worst	0.87(0.55–1.55)	6.22(2.77–6.41)	1.17(0.65–1.60)

Data are expressed as median values and interquartile range (25th, 75th percentile), or number. KD, Kawasaki disease; CAL, coronary artery lesion; Illness days, illness days before sample collection; WBC, white blood cell; #N, absolute value of neutrophil; Plt, platelet; ESR, erythrocyte sedimentation rate; and CRP, C-reactive protein.

### Cell culture and preparation

Primary HCAECs (SCIENCELL, California, America) were obtained and cultured using an Endothelial Cell Medium (SCIENCELL, California, America) containing with 1% endothelial cell growth supplement (ECGS) and 5% fetal bovine serum (FBS). The culture medium was changed every 24 h. When HCAECs were 70–80% confluent, the cells were trypsinized, resuspended in the culture medium and seeded into 96-, 24-, or 6-well microplates for each assay. HCAECs were used for experiments between third and fifth passage. When HCAECs were 90% confluent, the medium was exchanged with endothelial cell basal medium-2 (SCIENCELL, California, America) overnight and then incubated with 15% sera from KD patients or healthy children. Subsequently, HCAECs were cultured in a 5% CO_2_ incubator for 6 h at 37 °C. All experiments were repeated at least three times.

### Cell counting kit-8

HCAECs were cultured in 96-well round-bottomed plates at a density of 3*10^3^ cells/well. Next day, the medium was exchanged with endothelial cell basal medium-2 with 15% human sera. Following 24 h and 48 h of culture, the medium was supplemented with 10 μL CCK (7SEA BIOTECH, Shanghai, China) for 4 h. The optical density (OD) at a wavelength of 450 nm was measured (MERINTON, Beijing, China). The complete medium was always used as an internal control in the assay, and the percent increase or decrease in cell proliferation relative to the internal control was calculated for each sample. Each sample was examined in triplicate and the mean and SD was calculated.

### Real-time cell electronic sensing assay

Complete medium (100 uL) containing 3*10^3^ HCAECs was loaded in each well of the 16-well plate. The plate was incubated for at least 30 min in a humidified (37 °C) 5% CO2 incubator and then was inserted into the real-time cell electronic sensing (RT-CES) system (ACEA BIOSCIENCES, Inc.). Next day, the medium was exchanged with endothelial cell basal medium-2 with 15% human sera.

### Endothelial cell tube formation assay

A total of 2 × 10^4^ HCAECs per well were plated into the surface of the polymerized ECMatrix glue (MILLIPORE, Billerica, MA, USA), incubated at 37 °C in 5% CO_2_ for 4 h in the presence of 15% pooled sera from either patients or healthy children, and photographed using an inverted phase contrast photo microscope at a magnification of 40× (NIKON E100). The tube formation was manually analyzed in each photo by IMAGE J. Each sample was examined in triplicate and the mean and SD was calculated.

### RNA isolation and reverse transcription-quantitative polymerase chain reaction (RT-qPCR)

Total RNA was isolated from cells using RNeasy Mini kit (QIAGEN, Dusseldorf, German) according to the manufacturer’s protocol. The extracted RNA was reverse transcribed into cDNA using PrimeScript RT Master Mix (TAKARA, Dalian, China). Real-time quantitative PCR analyses were performed using the Applied BIOSYSTEMS 7500 Real-Time PCR System with various sequences. All RT-PCR primers were from SYBR green and *GAPDH* was the reference gene. The sequences of the primer pairs were shown in Supplementary Table S1. mRNA levels were determined by the ΔΔCt relative quantitative analysis method.

### Western blot analysis

Briefly, the total proteins were prepared by RIPA buffer containing protease and phosphatase inhibitors (BEYOTIME INSTITUTE, Jiangsu, China). The cytoplasm proteins and nucleus proteins were extracted by Nuclear and Cytoplasmic Protein Extraction Kit (BEYOTIME INSTITUTE) according to the manufacturer’s protocol. Equal protein amounts were treated by SDS-PAGE electrophoresis, protein transfer electrophoresis, and nonfat milk blocking, and incubated to the primary antibody NFATc1 (dilution 1: 2,000; ABCAM, Cambridge, United Kingdom), NFATc3 (dilution 1: 2,000; ABCAM), VCAM-1 (dilution 1: 10,000; ABCAM), GAPDH (dilution 1: 10,000; ABCAM), b-tubulin (dilution 1: 2,000; CELL SIGNALING TECHNOLOGY, MA, USA) and HistoneH3 (dilution 1: 2,000; HUABIO, Hangzhou, China), respectively, overnight at 4 °C. Subsequently, the blot was incubated to horseradish peroxidase (HRP)-conjugated secondary antibodies (dilution 1: 10,000; ABCAM) for 1 h at room temperature, and was interacted with chemiluminescent substrate (THERMO SCIENTIFIC, MA, USA). Densitometry of bands was quantified by Image software (IMAGE J, National Institutes of Health) and expressed as the mean gray value. All western blot experiments were examined in triplicate and the mean and SD was calculated.

### Statistical analysis

Continuous variables are expressed as median values and interquartile ranges (IQR; 25th–75th percentiles). Categorical variables are presented as frequencies. For normally distributed data, values were expressed as means ± SEM. Student’s t test or Welch’s t test was used to analyze differences between groups. Statistical analyses were performed with GRAPHPAD PRISM 7.0 (GRAPHPAD Software, CA, USA). *P* < 0.05 was considered statistically significant.

## Supplementary information

Supplementary Information.
