# Supplementary material for: The role of Ca2+/NFAT in Dysfunction and Inflammation of Human Coronary Endothelial Cells induced by Sera from patients with Kawasaki disease
Source: Sci Rep. 2020 Mar 13;10:4706. doi: 10.1038/s41598-020-61667-y (PMC7069934; doi:10.1038/s41598-020-61667-y)

The role of Ca^{2+}/NFAT in Dysfunction and Inflammation of Human Coronary Endothelial Cells induced by Sera from patients with Kawasaki disease

Running title: Ca^{2+}/NFAT in KD sera induced HCAECs

Ying Wang [#], Jian Hu [#], Jingjing Liu [#], Zhimin Geng, Yijing Tao, Fenglei Zheng, Yujia Wang, Songling Fu, Wei Wang, Chunhong Xie, Yiying Zhang, Fangqi Gong*

[#] Contributed equally

Affiliations: Department of Cardiology, Children's Hospital, Zhejiang University School of Medicine, National Clinical Research Center for Child Health. No. 3333 Binsheng Road, Hangzhou, 310052, PR China.

Corresponding author: Fangqi Gong, Department of Cardiology, The Children's Hospital of Zhejiang University School of Medicine, National Clinical Research Center for Child Health. No.3333, Binsheng Road, Hangzhou 310052, PR China. Tel/Fax: +86-571-86670008, E-mail: gongfangqi@zju.edu.cn

Supplementary Table S1. The sequences of the primer pairs.

Name	Forward primer sequence	Reverse primer sequence
GAPDH	GGAGCGAGATCCCTCCAAAAT	GGCTGTTGTCATACTTCTCATGG
NFATc1	CACCGCATCACAGGGAAGAC	GCACAGTCAATGACGGCTC
NFATc3	TCCACCTCCATCTACTTTAACCA	TTGGGACCACCTAATGGGCT
E-selectin	CAGCAAAGGTACACACACCTG	CAGACCCACACATTGTTGACTT
P-selectin	ATGGGTGGGAACCAAAAAGG	GGCTGACGGACTCTTGATGTAT
VCAM-1	TTTGACAGGCTGGAGATAGACT	TCAATGTGTAATTTAGCTCGGCA
ICAM-1	ATGCCCAGACATCTGTGTCC	GGGGTCTCTATGCCCAACAA
TF	CCCAAACCCGTCAATCAAGTC	CCAAGTACGTCTGCTTCACAT
MCP-1	CAGCCAGATGCAATCAATGCC	TGGAATCCTGAACCCACTTCT
IL-8	ACTGAGAGTGATTGAGAGTGGAC	AACCCTCTGCACCCAGTTTTC

Supplementary Figure S1: Full-length blots

Full-length blots for Figure 1

b

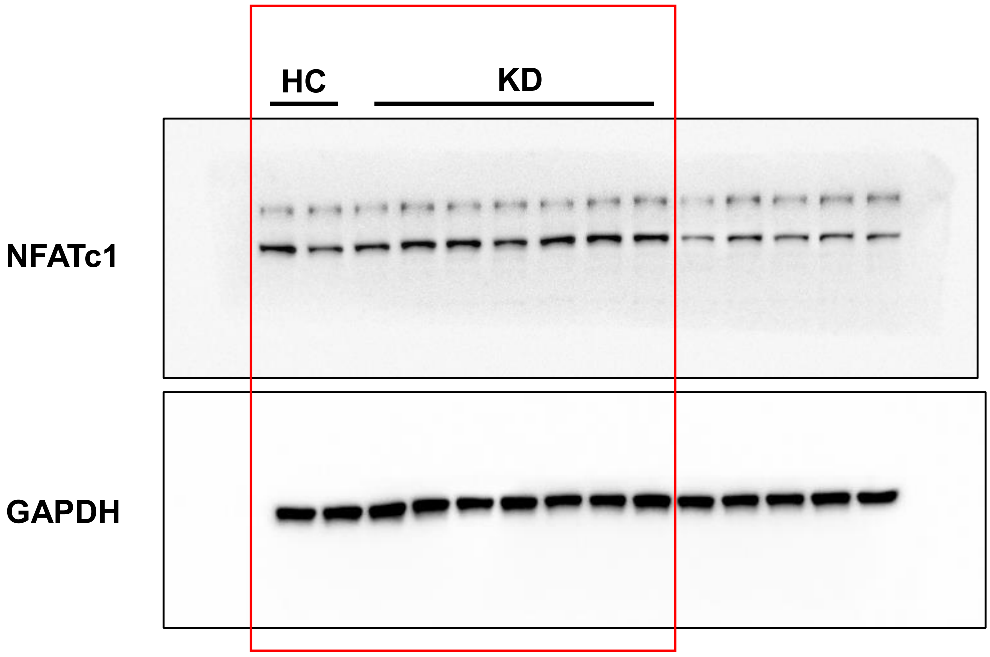

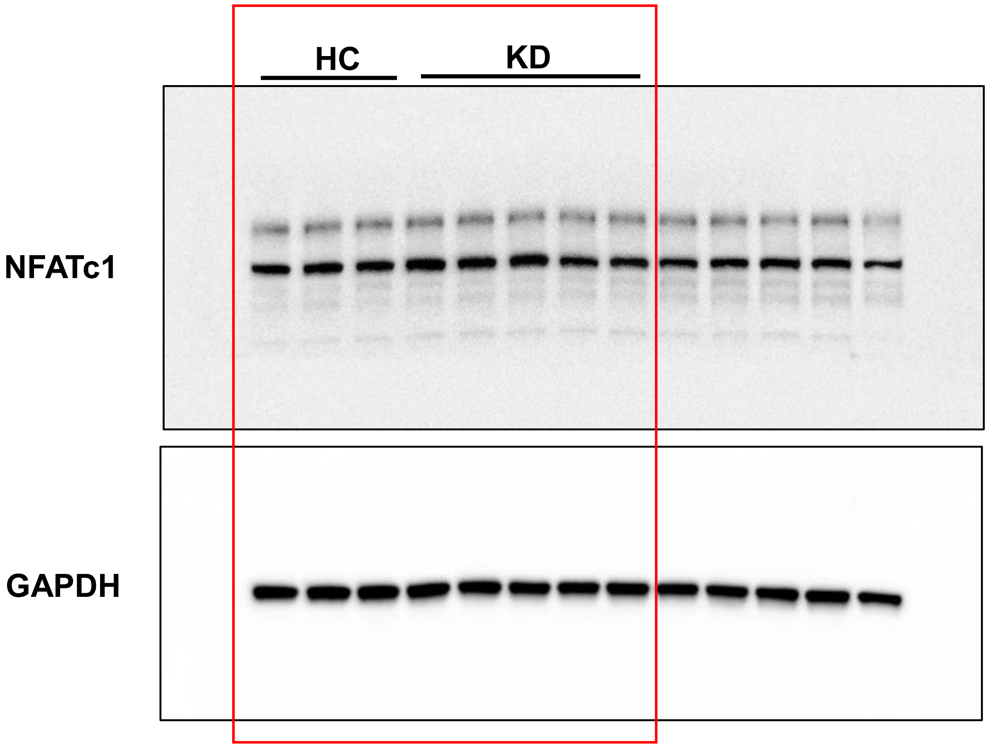

Full-length blots for Figure 2

a

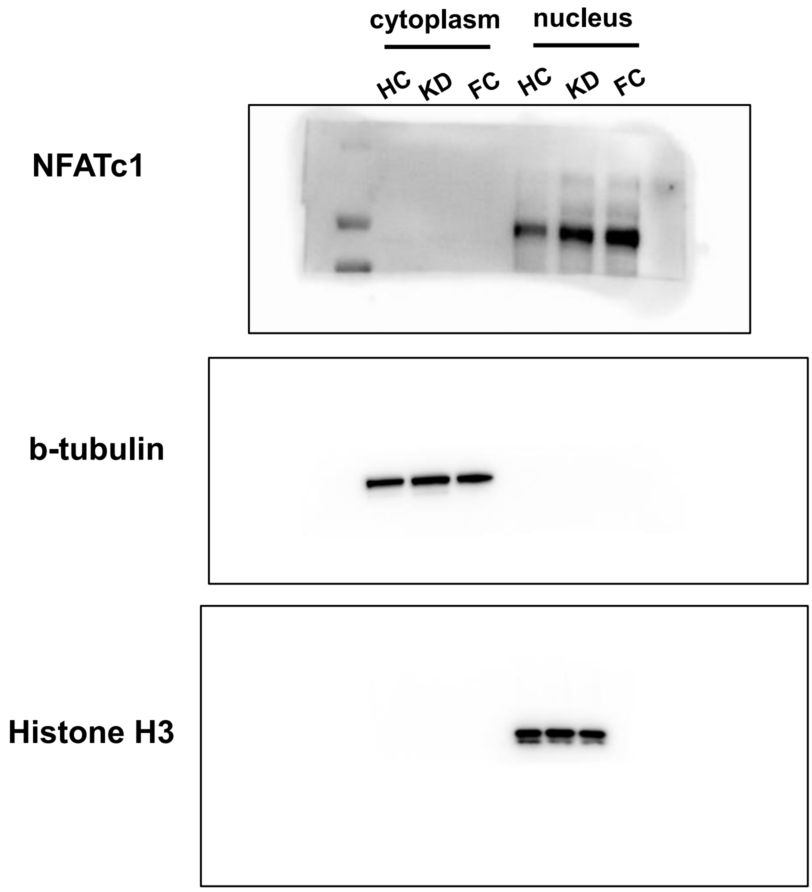

b

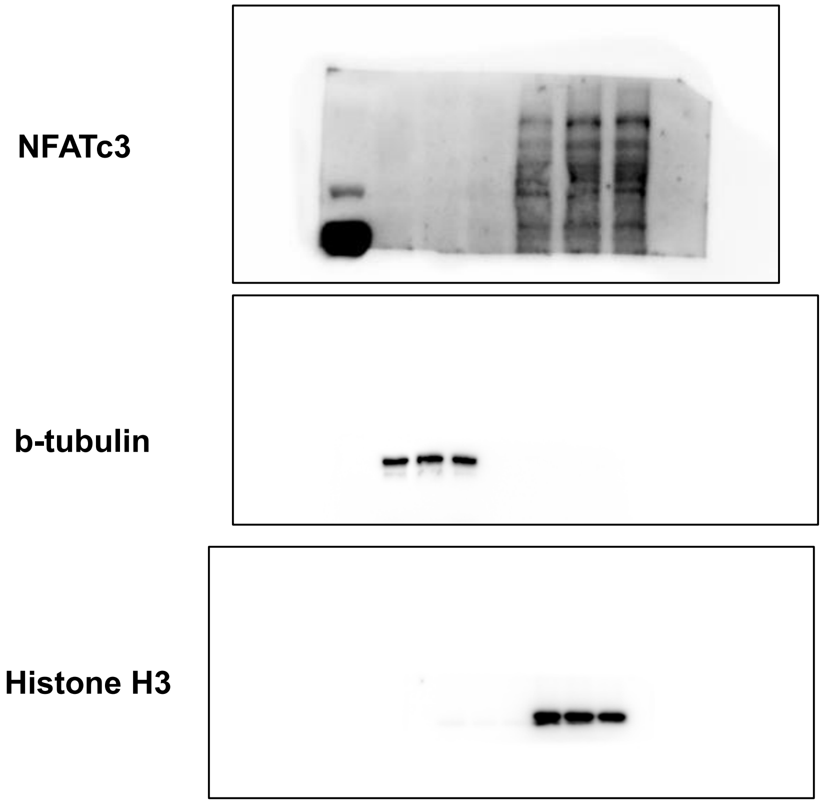

Full-length blots for Figure 4

b

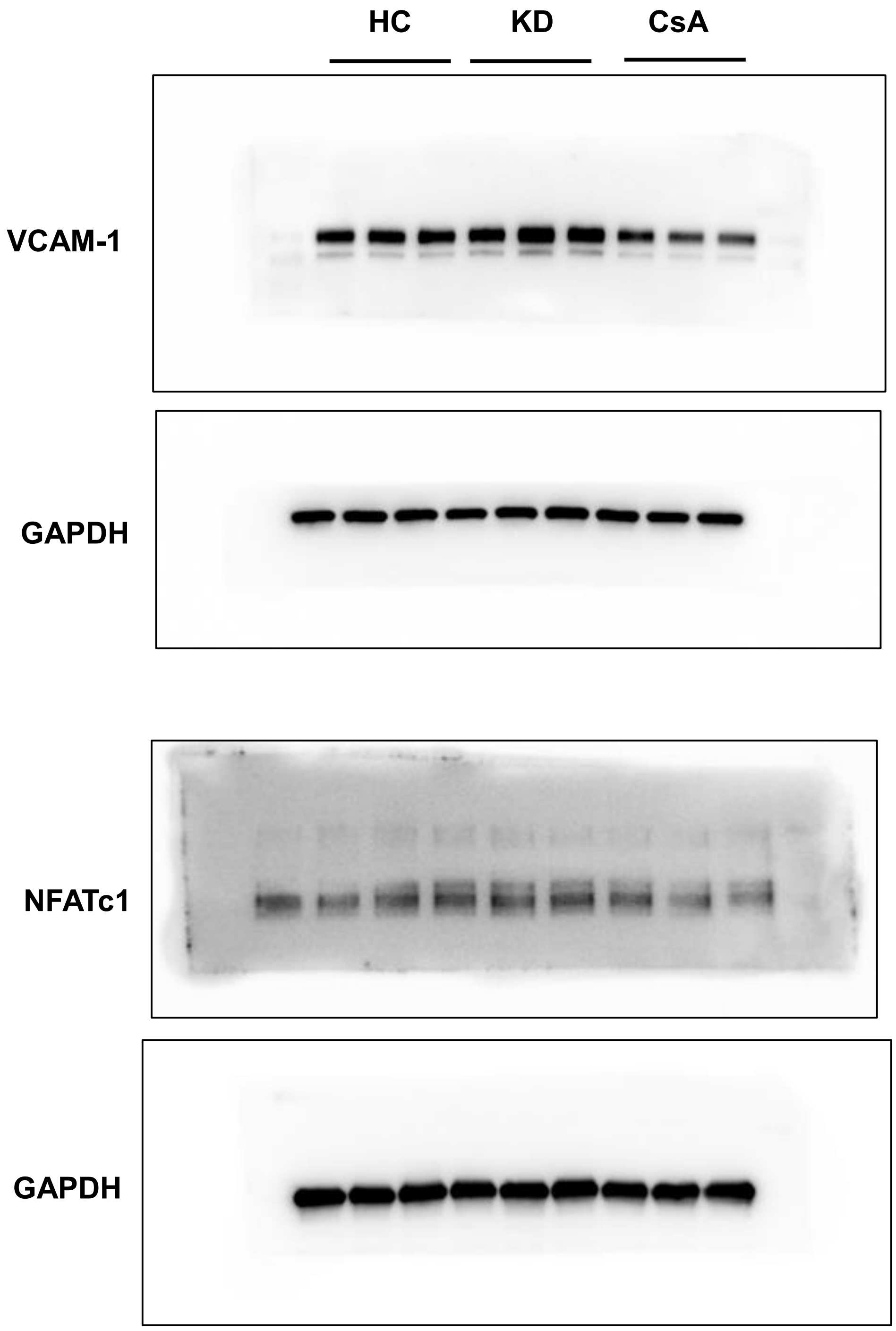

Supplementary Figure S2: Multiple exposures of Figure 2 b-tubulin and Histone H3 blots

Fig. 2a b-tubulin:

Exposure time

5s:

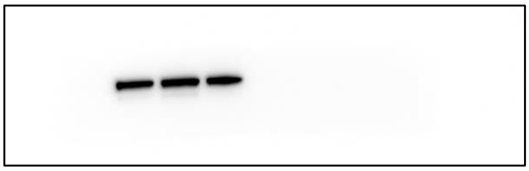

7s:

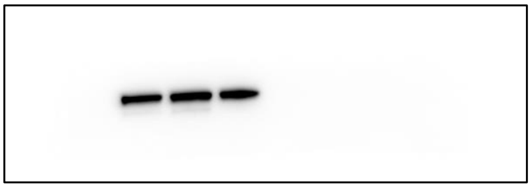

Fig. 2b b-tubulin:

Exposure time

3s:

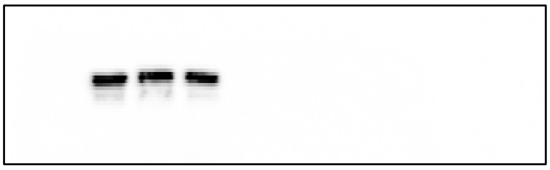

5s:

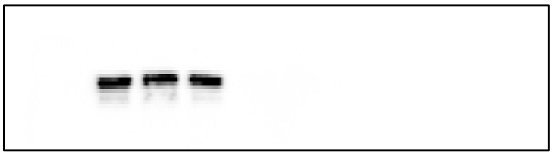

10s:

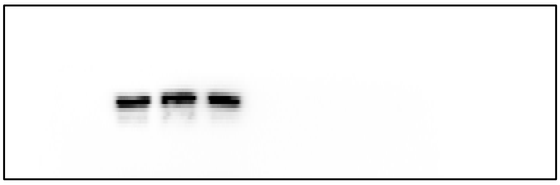

Fig. 2a Histone H3:

Exposure time

0.5s:

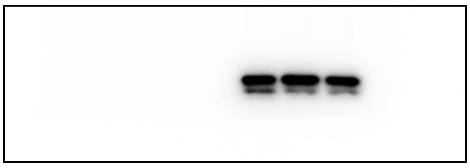

1s:

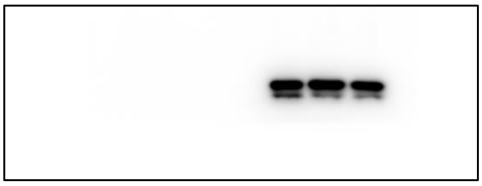

2s:

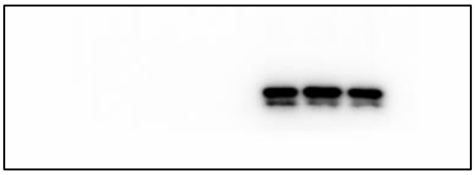

3s:

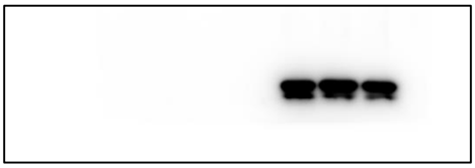

5s:

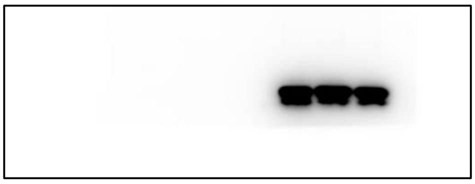

Fig. 2b Histone H3:

Exposure time

0.5s:

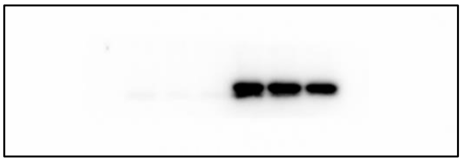

1s:

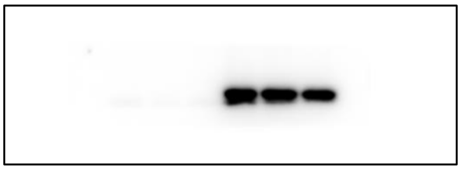

2s:

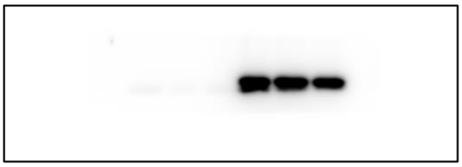

5s:

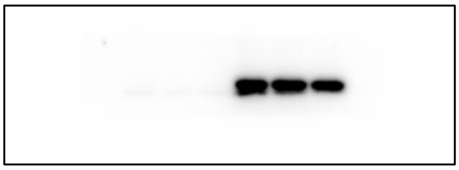

10s:

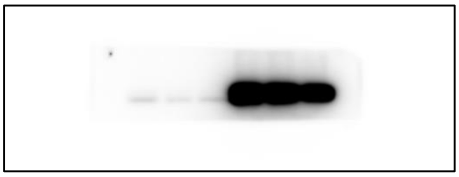

Supplement: Supplementary file 1 — Supplementary Information. [file 41598_2020_61667_MOESM1_ESM.pdf]
